# The Structure of Academic Self-Concept When Facing Novel Learning Content: Multidimensionality, Hierarchy, and Change

**DOI:** 10.5964/ejop.v15i3.1716

**Published:** 2019-09-27

**Authors:** Julia Gorges, Jelena Hollmann

**Affiliations:** a Department of Psychology, Educational Psychology, Bielefeld University, Bielefeld, Germany; University of Wroclaw, Poland

**Keywords:** academic self-concept, self-concept structure, higher education, motivation, confirmatory factor analysis

## Abstract

Academic self-concepts of ability are key factors in promoting education and learning throughout students’ school career. Yet we know little about their structure and structural change when students leave high school to face novel academic tasks. The present study investigated the structure and structural change of first-year students’ study-related self-concepts of ability. Data stems from a longitudinal study with two measurement points covering the initial study phase (t1: N = 341; age: M = 21.6; SD = 3.56; 57.5% female). Self-concepts were assessed regarding the participants’ study program and four of its subordinate subjects. Confirmatory factor analyses and structural equation models were used to compare structural models and to investigate structural stability and directional effects. Results support the assumption of multidimensionality (i.e., distinct self-concepts for different subjects) and hierarchy with a generic field-of-study-specific self-concept at the apex. Specifying generic field-of-study-specific self-concept as a method factor (i.e., indicated by both subject-specific and field-of-study-specific items) was most consistent with theoretical assumptions. The structural model was invariant over the first months at university. Generic field-of-study-specific self-concept and subject-specific self-concepts largely developed independently from one another. The results emphasize the recently suggested conceptualization of generic self-concept as a method factor to reflect self-concept hierarchy. Self-concepts were structurally stable over time. Several significant horizontal effects (i.e., stability within subjects) suggest that students align their self-concepts closely to the curriculum they encounter in educational contexts and, therefore, may benefit from ample feedback on their performance to develop appropriate subject-specific self-concepts.

Ample research has documented the significance of ability perceptions for individuals’ well-being and success in many different contexts such as education (Marsh, 2007), work (Leonard, Beauvais, & Scholl, 1999), and daily life in general (Elliot & Dweck, 2005; see Marsh, Martin, Yeung, & Craven 2017, for a recent review). The conceptualization of academic self-concept of ability in particular stems from research on self-esteem and self-views in different life domains. Hence, researchers distinguish an individual’s emotional, social, and academic self-concept, for example (Marsh & Craven, 2006). The academic self-concept became very prominent in educational psychological research because academic self-concepts predict students’ self-esteem, motivation, task choice, and achievement in academic contexts (cf. Marsh, 2007; Marsh et al., 2017). Academic self-concepts of ability—that is to say, students’ beliefs about what they can and cannot do in academic contexts—are key factors in promoting education and learning (cf., Marsh, 2007; Marsh et al., 2017).

Self-concept research has mostly focused on high school students’ development and achievement. Theoretical models of academic self-concept posit a multidimensional and hierarchical structure with a generic academic self-concept enthroned at the apex of a range of subject-specific self-concepts (Byrne & Gavin, 1996; Shavelson, Hubner, & Stanton, 1976). Strong evidence supports the idea that self-concept is highly subject-specific (Marsh, 1992; Marsh, Hey, Johnson, & Perry, 1997; Yeung, Chui, & Lau, 1999; Vispoel, 1995). By contrast, evidence for its hierarchical nature is less consistent (Brunner et al., 2010; Marsh, 1990; Yeung et al., 1999; Yeung et al., 2000). Related to the question of hierarchy, the question of structural development—i.e., whether a generic academic self-concept develops from subject-specific self-concepts or vice versa—has likewise rarely been addressed (but see Marsh & Yeung, 1998). Finally, only a few studies have explicitly focused on self-concept structure and development when learners face novel academic tasks outside the high school curriculum, for example, in higher education (Tempelaar, Gijselaers, van der Loeff, & Nijhuis, 2007; Yeung et al., 1999).

Against this background, the present study seeks to extend the scarce research on the structure and structural change of academic self-concept when learners face novel academic tasks using two waves of data from first-year students in Germany. First, drawing on Brunner et al.’s (2010) work, we tested different structural models of self-concept, which have been primarily derived from high school contexts. Second, we investigated the structural stability of students’ self-concepts during the initial study phase. Third, we scrutinized Marsh and Yeung’s (1998) findings regarding directional effects between generic and subject-specific self-concepts over time.

## Multidimensionality of Academic Self-Concept

The Shavelson et al. (1976) self-concept model posits that academic self-concepts are tied to specific learning contents—typically high school subjects—and that these specific self-concepts are subordinate to a generic academic self-concept conceptualized as second-order factor. This assumption has been supported by a range of studies set within a primary or secondary school context, which operationalized self-concept specifically to school subjects (e.g., Byrne & Gavin, 1996; Marsh, 1992). Similarly, self-concepts may be specific to different facets of skills or subjects (e.g., languages; Arens & Jansen, 2015; Lau, Yeung, Jin, & Low, 1999; Yeung et al., 2000; creative arts; Vispoel, 1995; sports; Marsh, Hey, Johnson, & Perry, 1997). Focusing on a specific study program or educational context, Yeung et al. (1999) and Tempelaar et al. (2007) reported distinct self-concepts regarding subjects taught in higher education, such as accounting and economics. Extending the assessment of self-concept beyond labels of school subjects, Jansen, Schroeders, Lüdtke, and Pant (2014) found differential self-concepts for biology, chemistry, and physics in a sample of secondary school students who only have been taught science instead of biology, chemistry, and physics as distinct subjects. Similarly, Gorges and Göke (2015) report good discriminate validity for first-year students’ self-concepts with respect to four fields of study, only one of which the students were going to study.

These findings led us to believe that self-concepts typically reflect the learning content structure of students’ educational context. Differential experiences as well as labels attached to different areas of experience appear to be key factors that enable specific self-concept development even beyond curriculum areas. Thus, when respondents know which past experiences—achievements, failure, and affect—or future tasks—mathematical or verbal, for example—they are supposed to look at, they apparently are able to verbalize a self-concept for their ability in this area.

## Hierarchical Structure and the Conceptualization of Generic Academic Self-Concept

Research on the hierarchical nature of self-concept is scarce, and results have been mixed. Most prominently, a lack of correlation between subject-specific self-concepts from the mathematical versus verbal domain led Marsh to develop a revised structural model (1990) distinguishing two second-order factors (known as the Marsh/Shavelson model). Transcending high school subjects, research on self-concept hierarchy has focused on specific domains or content areas (e.g., creative arts; Vispoel, 1995; language; Arens & Jansen, 2015; Lau et al., 1999; Yeung et al., 2000), or specific educational contexts. In fact, Yeung et al. (1999) suggested that the assumption of hierarchy only holds in educational contexts focusing on one particular content domain because the high school curriculum covers a too diverse range of topics to build a hierarchal structure of subject-specific self-concepts. Consequently, the authors investigated self-concepts of students at a school of commerce, covering subject-specific self-concept for math/statistics, economics, accounting, English, and Chinese. Generic academic self-concept has been measured with reference to the students’ current educational context (“perception in most subjects,” p. 379). Results support the hypothesized structure. Comparing conceptualizations of the second-order factor as a latent variable based on the specific first-order factors versus a latent variable based on manifest items referring to a generic academic self-concept shows that both ways of conceptualizing generic (academic) self-concept overlap substantially (*r* > .90) when parallel self-concept items on both hierarchical levels were used (Arens & Jansen, 2015; Yeung et al., 2000).

**Figure 1:**
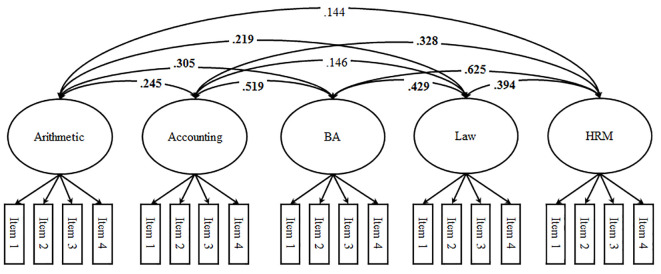
First-order factor model (standardized loadings and correlations). *Note*. BA = business administration (i.e., field of study); HRM = human resources management; significant correlations (*p* < .05) are printed in bold, factor loadings and correlated uniqueness are not depicted in the figure.

**Figure 2:**
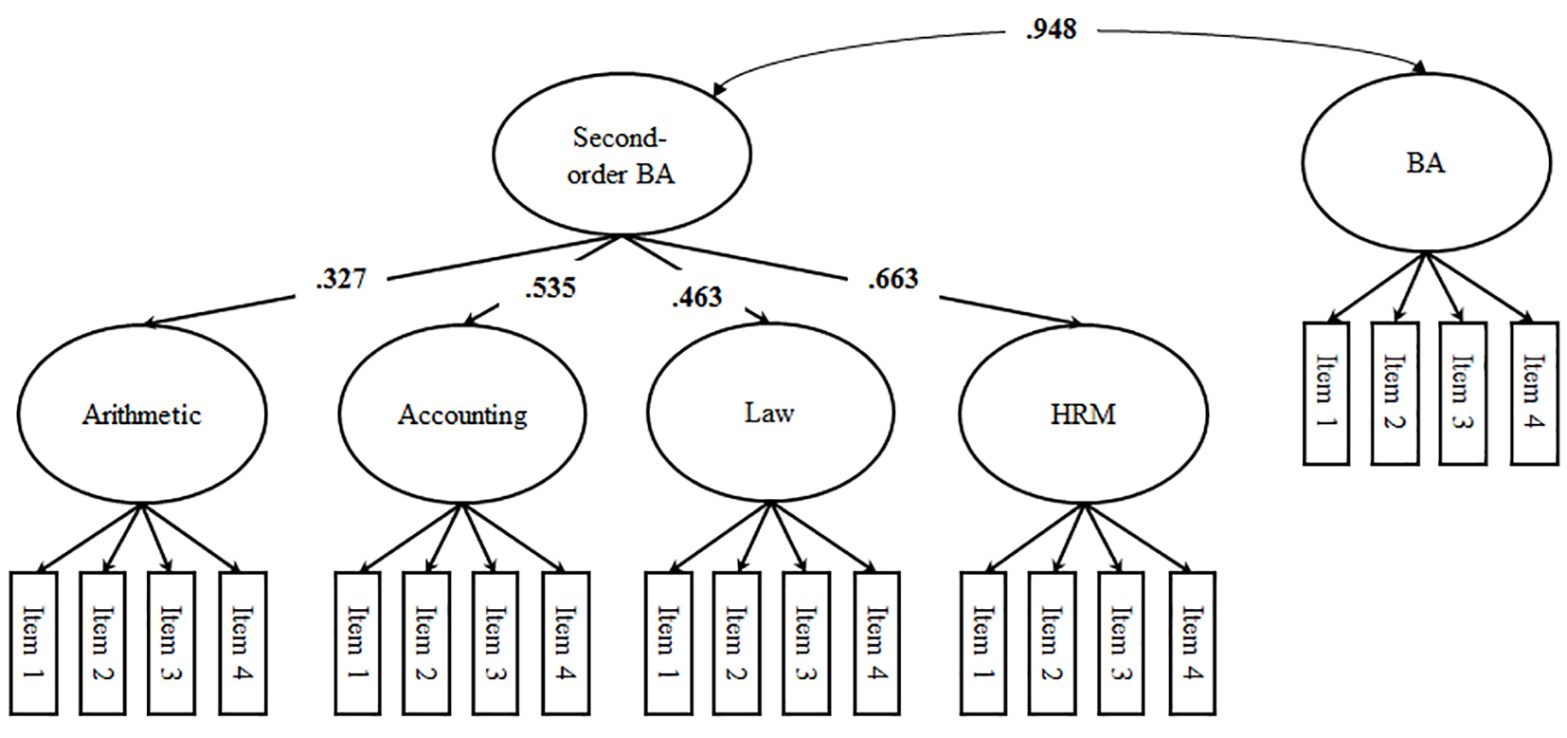
Shavelson Model with a correlated first-order factor reflecting the field-of-study (i.e., business administration)-specific self-concept (standardized loadings and correlations). *Note*. BA = business administration (i.e., field of study); HRM = human resources management; significant correlations (*p* < .05) are printed in bold, factor loadings and correlated uniqueness are not depicted in the figure.

**Figure 3:**
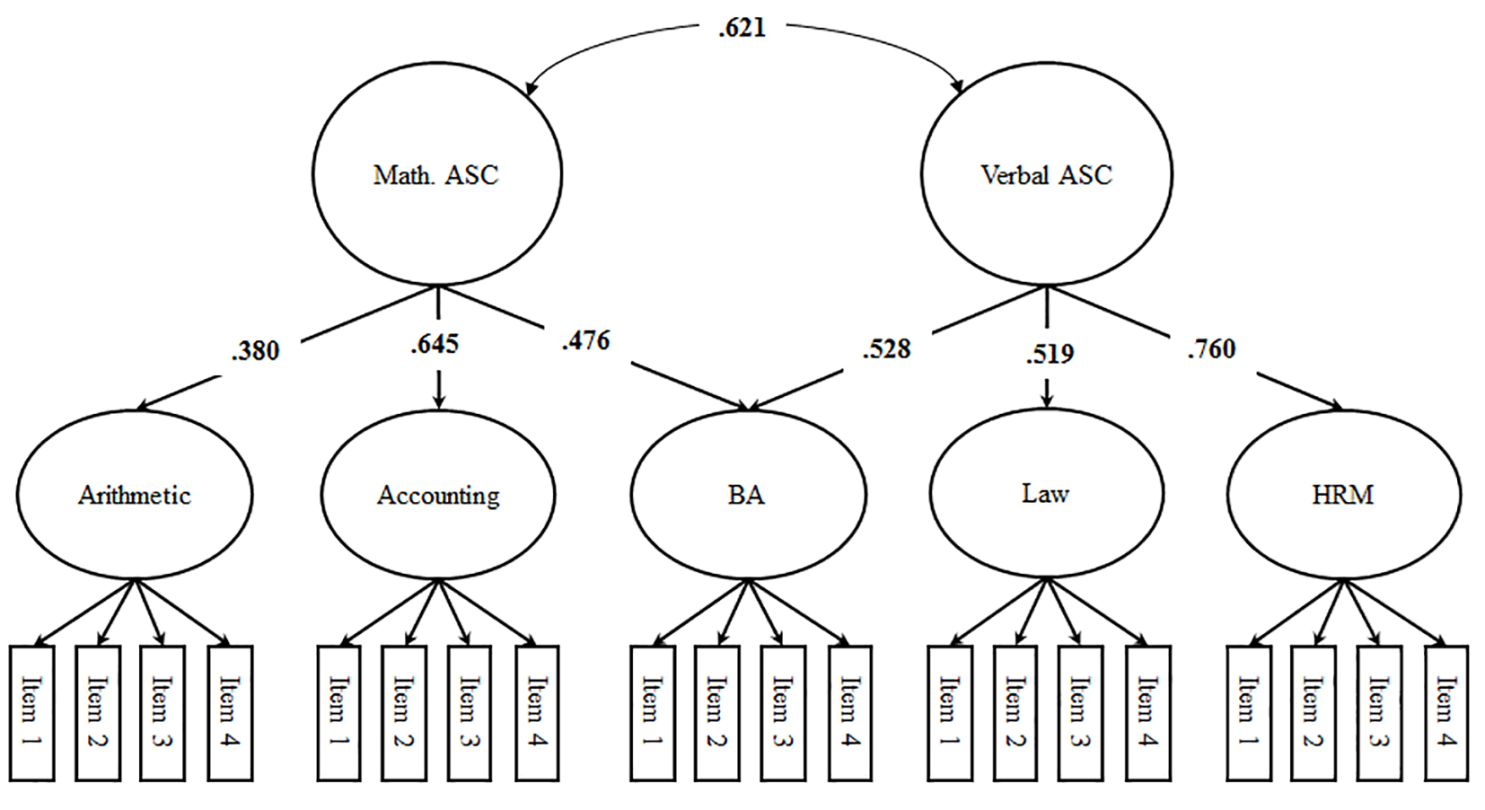
Marsh/Shavelson Model (standardized loadings and correlations). *Note*. ASC = academic self-concept; BA = business administration (i.e., field of study); HRM = human resources management; significant correlations (*p* < .05) are printed in bold, factor loadings and correlated uniqueness are not depicted in the figure.

**Figure 4:**
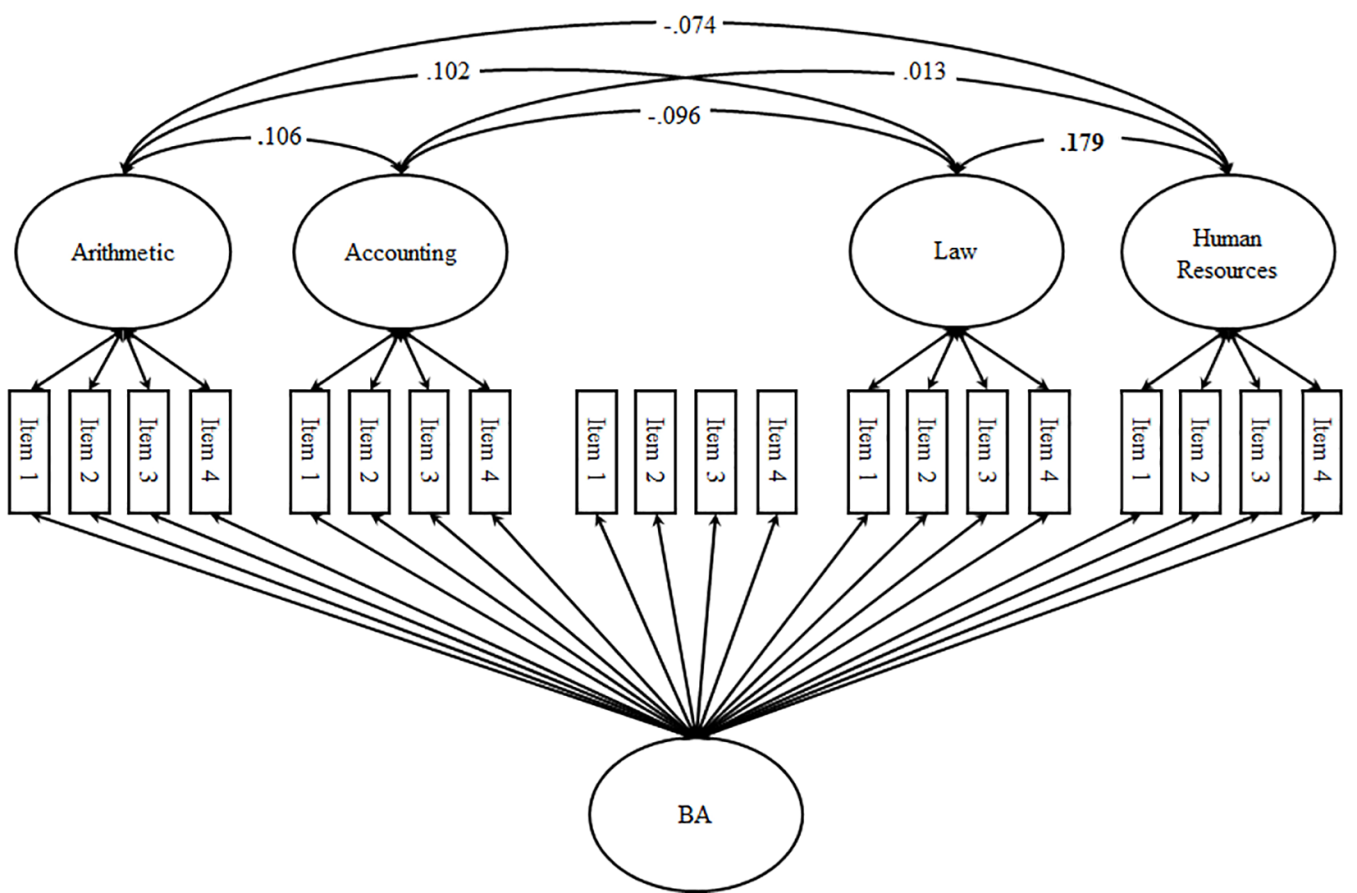
Nested Marsh/Shavelson Model (standardized loadings and correlations). *Note*. BA = business administration (i.e., field of study); significant correlations (*p* < .05) are printed in bold, factor loadings and correlated uniqueness are not depicted in the figure, for factor loadings and factor correlations, see Table 2 and Table 3, respectively.

Reconciling the three theoretical predictions made by Shavelson et al. (1976)—multidimensionality and hierarchy with a generic academic self-concept at the apex of the hierarchy—and inconsistent findings from empirical studies, Brunner et al. (2010) proposed a nested Marsh/Shavelson model, which accounts for the assumptions of multidimensionality and hierarchy, with only one superordinate generic academic self-concept. Drawing on confirmatory factor analytic approaches that enable researchers to differentiate between variance attributed to latent constructs and variance attributed to method factors (Eid, Lischetzke, Nussbeck, & Trierweiler, 2003), they conceptualized generic academic self-concept as a method factor. Hence, the model differentiates between subject-specific portions of variance in self-concepts (first-order factors) and portions that reflect a person’s generic academic self-concept drawing on both subject-specific items and items that tap generic academic self-concept directly (method factor; see Figure 4). Comparative analyses of a first-order factor model with correlated factors, a Shavelson model, a Marsh/Shavelson model (see Figures 1–3), and their nested Marsh/Shavelson model supported Brunner et al.’s structural model. Hence, the hierarchical nature of self-concept may best be reflected by specifying a generic academic self-concept as a method factor.

## Structural Changes of Self-Concepts

Self-concepts of ability generally develop from students’ experience within the respective content domain (cf. Marsh, 2007) resulting in increasingly stable self-concepts (Denissen, Zarrett, & Eccles, 2007; Musu-Gillette, Wigfield, Harring, & Eccles, 2015), including structural stability (Byrne & Gavin, 1996). Focusing on longitudinal within-person development, of particular interest is the relation between generic academic and subject-specific self-concepts. Testing directional effects based on two datasets using different self-concept measures, Marsh and Yeung (1998) found support for neither top-down effects (i.e., generic self-concept affects subject-specific self-concepts) nor bottom-up effects (i.e., subject-specific self-concepts affect generic self-concept). Rather, both subject-specific and generic academic self-concepts appear to be stable over time, with little to zero cross-lagged effects, but substantial horizontal effects (i.e., effects over time within the respective level of abstraction).

It has to be noted, however, that the data used by Marsh and Yeung (1998) stemmed from adolescent high school students with presumably highly stable subject-specific self-concepts (Denissen et al., 2007; Musu-Gillette et al., 2015). Horizontal effects—that is, stability coefficients—unsurprisingly are stronger than top-down or bottom-up effects that indicate some variability. Given that self-concepts rely on personal experience with a content or domain, variability in self-concept should be particularly pronounced when students face novel contents or domains (Gorges, 2017). Hence, changes should become observable when students build new self-concept structures. With this in mind, the present study draws on a sample of first-year students who have been surveyed about their academic self-concepts right at the beginning of the study program (i.e., when the field of study constitutes a novel learning content), and toward the end of their first semester. One may argue that such anticipated self-concepts prior to the beginning of students’ study program rather constitute self-efficacy beliefs because they are future-oriented (Bong & Skaalvik, 2003). However, we focus on aggregated and presumably hierarchically structured ability beliefs tied to a general field of study or to a subordinate subject, respectively, rather than a specific task. Furthermore, previous studies have shown that self-concepts regarding novel academic tasks rely on school subject-specific ability beliefs (Gorges & Göke, 2015). Hence, anticipated academic self-concepts considering future learning contents are still past-oriented.

## The Present Study

The present study investigated the basic assumptions of self-concept structure and its structural development. The first goal of this study is to extend the scarce research on self-concept structure when learners face novel academic learning content. We deliberately focused on a study program encompassing a wide array of curriculum content areas (i.e., business administration). We investigated multidimensionality and hierarchy based on the four structural models outlined by Brunner et al. (2010): the first-order factor model, the Shavelson model, the Marsh/Shavelson model, and the nested Marsh/Shavelson model. Unlike previous studies, we postulated generic field-of-study-specific self-concept—and not generic academic self-concept—to be at the apex of the hierarchy. Second, we assumed that first-year students initially develop ability perceptions regarding their field of study as a whole, which is typically associated with mathematics (Gorges & Göke, 2015). With time and personal experience, students should discover that their field of study is associated with a range of different subject matters, some of which are rather similar to mathematics (e.g., business arithmetic, accounting) and some of which are not (e.g., human resources, law). Accordingly, we assumed that—prior to beginning their study program—first-year students’ anticipated self-concept would be less differentiated and focused on mathematical components. By contrast, relations between verbal subject-specific self-concepts (i.e., law and human resources) and a generic field-of-study-specific self-concept would be weaker but increase over time (see Gorges, 2017, on the revision of motivational beliefs). Third and finally, we investigated potential directional effects between field-of-study-specific and subject-specific self-concepts.

## Method

### Participants and Procedure

Participants were first-year students enrolled in study programs labeled *business administration* (in German “Betriebswirtschaft” or “Betriebswirtschaftslehre”) at one of six cooperating universities of applied sciences in Germany participating in the research project 'ValCom' (Values and Competencies in Adulthood). Online data collection took place during the weeks preceding the first semester up to the first weeks after the start of the study program (t1) and 3–4 months afterwards (i.e., toward the end of the first semester; t2). A participant-generated code linked the data across the two waves of data collection.

Overall, 408 students started the survey (response rate: 34%). All participants were informed about the purposes for which their data will be used and gave their consent. We excluded participants with missing data on all variables, and participants for whom codes and sociodemographic information could not be recorded due to technical problems. The final sample contained *n*_t1_ = 341 students (age: *M (SD)* = 21.6 (3.56); 57.5% female). Of these participants, 57% did not take part in wave two (*n*_t2_ = 195; *M (SD)* = 21.4 (3.55); 63.5% female).

### Measures

We used four items adapted from the literature (Dickhäuser, Schöne, Spinath, & Stiensmeier-Pelster, 2002) and parallel wording to tap students’ academic self-concepts for different learning contents. We asked for students’ self-concept for arithmetic, law, accounting, and human resources indicating non-mathematical basic economics in all but one curricula. Each item included a blank (“…”) and participants were instructed to fill this blank with the respective learning content stated on the survey page. Thus, the item “I consider my aptitude for ‘…’ to be high” could mean “I consider my aptitude for business administration to be high”, or “I consider my aptitude for human resources to be high”, and so on.

We used the same items and the same mode of presentation at both measurement points. To have the same subject label across all participating universities, we used the same major headings, but gave examples tailored to the specific curriculum. Participants were explicitly asked to anticipate their self-concept regarding study-related learning contents at t1. Items were presented in terms of a matrix where business administration shared a survey page with three more fields of study and the four subjects were presented together on the following survey page. Answers to all items were recorded using a four-point Likert-type scale (1 = *absolutely not true*, 2 = *rather not true*, 3 = *somewhat true*, 4 = *absolutely true*). Internal consistency of all scales was high (α > .80).

### Analyses

We started our analyses by performing MANOVAs and χ^2^-Tests to see whether dropout between t1 and t2 would bias the findings. To address the structure of students’ self-concepts we specified four structural models using t2 data: the first-order factor model (Model 1; see Figure 1), the Shavelson model alongside a first-order-factor reflecting business administration-specific self-concept (Model 2; see Figure 2), the Marsh/Shavelson model (Model 3; see Figure 3), and the nested Marsh/Shavelson model (Model 4; see Figure 4). We accounted for parallel items by specifying correlated uniqueness in all models (Marsh, Byrne, & Craven, 1992). We compared the four models based on fit indices, factor loadings, and factor correlations. In addition, we examined how closely the models reflect the theoretical assumptions of multidimensionality and hierarchy with a generic field-of-study-specific self-concept at the apex of the hierarchy. We moved on using the best fitting model.

With respect to our second goal, we investigated structural stability by specifying the same structural model for t2 and t1 data. Again, correlated uniqueness was specified within each measurement point. Next, we tested structural invariance by successively fixing factor loadings, intercepts, and residual variances to be equal across measurement points (i.e., testing weak, strong, and strict invariance; Meredith, 1993). In addition, we inspected changes in factor correlations.

**Figure 5:**
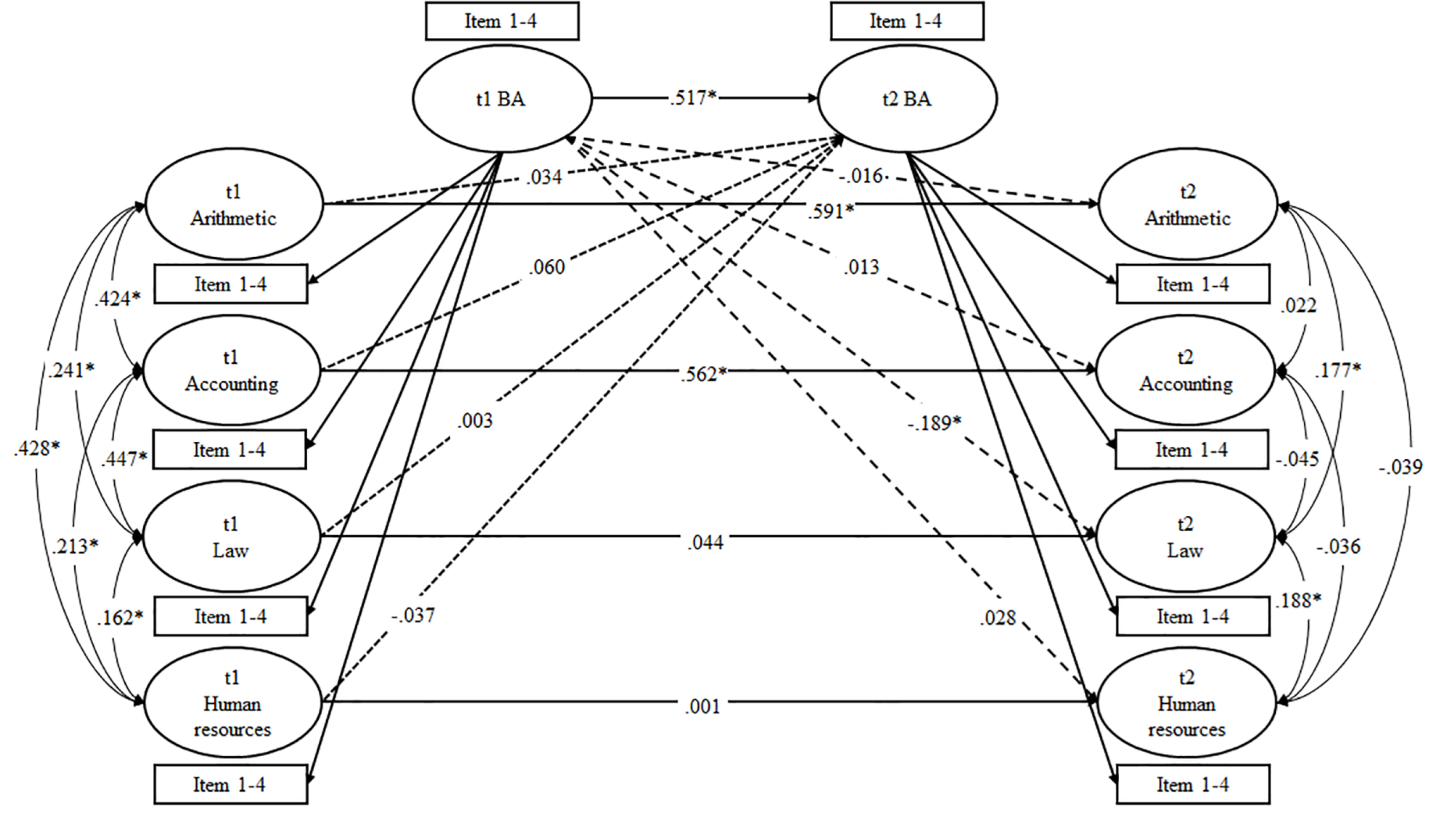
Structural development (standardized loadings and correlations). *Note*. BA = business administration (i.e., field of study); * *p* < .05; single items and correlated uniqueness are not depicted to increase clarity of the figure (see Figure 4 for a detailed depiction of the structural model), broken lines reflect top-down effects (Model 7), dotted lines represent bottom-up effects (Model 8), and solid lines reflect horizontal effects (Model 5, 6 and 9).

Finally, we replicated the structural development models proposed by Marsh and Yeung (1998). Our first model included horizontal effects for generic field-of-study-specific self-concept only (Model 5). Next, we added horizontal effects of subject-specific self-concepts (Model 6). In Model 7, we tested top-down effects only, whereas Model 8 contained bottom-up effects only. Finally, we tested a model including top-down, bottom-up, and horizontal effects simultaneously (Model 9, see Figure 5).

Mplus 7.11 (Muthén & Muthén, 1998–2016) was used to fit the data to the specified models in all analyses. To make use of all available data for model estimation, we used the full information maximum likelihood estimation (FILM; Schafer & Graham, 2002). We evaluated model fit based on the following fit indices: the Akaike information criterion (AIC; the smaller the better), the comparative fit index (CFI, good >.95; acceptable > .90), the root mean square error of approximation (RMSEA; good <. 05; acceptable < .08), and the standardized root mean residual (SRMR; acceptable < .10; good < .05; Schermelleh-Engel, Moosbrugger, & Müller, 2003). To compare nested models in tests of structural invariance, we considered a decrease in CFI > .01 and an increase in RMSEA > .015 as indicators of significant changes in model fit (Chen, 2007; Cheung & Rensvold, 2002).

## Results

### Dropout Analyses

Drop-out analyses revealed that the proportion of females was significantly higher at t2 (*p* < .05). However, MANOVA indicates no main effect of dropout on the t1 variables (*p* = .094). Thus, attrition does not appear to bias the findings.

### Structure of Students’ Self-Concept

As expected, all structural models provided an acceptable fit to the data (see Table 1, Models 1–4). Fit-indices were within a rather small range, indicating comparable fit across the four models. Model 1 (first-order factor model) supported the assumption that academic self-concepts of university students are subject-specific by showing substantial factor loadings across all first-order factors (all λ > .69, *p* < .05) and moderate factor correlations. The pattern of correlations suggested that subject-specific self-concepts contain a substantial proportion of shared variance. In addition, verbal and mathematical self-concepts, respectively, correlated more strongly within than across domains (see Figure 1)

**Table 1:** Model Fit of Alternative Structural Models (1–4) and for Longitudinal Models (5–9)

Model	*χ^2^*	*df*	AIC	CFI	RMSEA	95% CI RMSEA	SRMR
1	227.993*	120	5152.552	0.963	0.068	[0.054, 0.081]	0.047
2	237.664*	125	5152.223	0.961	0.068	[0.055, 0.081]	0.061
3	232.380*	123	5150.939	0.963	0.068	[0.054, 0.081]	0.055
4	206.091*	108	5154.650	0.966	0.068	[0.054, 0.082]	0.040
5	1316.975*	666	15828.459	0.921	0.054	[0.049, 0.058]	0.106
6	1207.146*	662	15726.630	0.934	0.049	[0.045, 0.054]	0.073
7	1356.990*	663	15874.474	0.916	0.055	[0.051, 0.060]	0.141
8	1359.129*	663	15876.613	0.915	0.056	[0.051, 0.060]	0.135
9	1200.590*	654	15736.074	0.934	0.050	[0.045, 0.054]	0.071

*Note.* Model 1 = first order factor model; Model 2 = one latent second-order factor (Shavelson model) correlated with a first-order generic BA factor; Model 3 = two higher-order factors indicating mathematical and verbal self-concept (Marsh/Shavelson model); Model 4 = first order factors and generic business administration self-concept (SC) as method factors (nested Marsh/Shavelson model); Model 5 = horizontal effect of generic business administration SC; Model 6 = Model 5 with additional horizontal effects of subject-specific SCs; Model 7 = top-down effect of t1 generic business administration SC on t2 subject-specific SCs, Model 8 = bottom-up effects of t1 subject-specific SCs on t2 generic business administration SC; Model 9 = top-down, bottom-up, and horizontal effects; χ^2^ = chi-square goodness-of-fit, df = degree of freedom; AIC = Akaike information criterion; CFI = comparative fit index; RMSEA = root mean square error of approximation; SRMR = standardized root mean square residual.

*p < .05.

In Model 2—the Shavelson model—first-order factor loadings were almost identical to those in Model 1. Factor loadings of the second-order factor are depicted in Figure 2. The second-order factor correlated almost perfectly with the first-order generic field-of-study-specific self-concept factor indicated by field-of-study-specific items.

In Model 3, subject-specific first-order factors were well defined with almost identical factor loadings to those in Model 1. Mathematical and verbal self-concept correlated substantially (*r* = .621, *p* < .05), and second-order factor loadings supported the assumption that the field of study, business administration, covers both verbal and mathematical subjects (see Figure 3). Compared to the Shavelson model, loadings on the domain-specific higher-order factors were somewhat higher. The two verbal subjects, law and human resources, show higher loadings on a domain-specific verbal self-concept compared to a generic field-of-study-specific self-concept factor. However, the correlation between verbal and mathematical self-concept still suggests substantial shared variance.

First-order factor loadings in Model 4—the nested Marsh/Shavelson model (Brunner et al., 2010)—are summarized in Table 2. Results revealed substantial factor loadings for all first-order subject-specific self-concepts (all λ > .597; *p* < .05). For the generic field-of-study-specific self-concept factor, factor loadings ranged between λ = .222 and λ = .847 (all *p* < .05). In addition to factor loadings for items referring to the field of study directly (all λ > .739; all *p* < .05), loadings for items referring to the subject arithmetic were lowest, whereas items referring to the subject human resources were highest. In line with theoretical assumptions, the generic field-of-study-specific factor captured variance shared by the first-order factors indicated by markedly lower and mostly non-significant factor correlations (see Figure 4).

**Table 2:** Standardized Factor Loadings Obtained in Model 4 (Nested Marsh/Shavelson Model) for t1 and t2

Item	(1)		(2)		(3)		(4)		(5)	
	t1	t2	t1	t2	t1	t2	t1	t2	t1	t2
(1) Business administration										
BA 1	.786	.780								
BA 2	.769	.847								
BA 3	.787	.796								
BA 4	.756	.739								
(2) Arithmetic										
A 1	.268	.250	.718	.801						
A 2	.350	.289	.585	.911						
A 3	.350	.325	.847	.892						
A 4	.256	.222	.825	.866						
(3) Accounting										
Acc 1	.416	.398			.421	.705				
Acc 2	.545	.437			.670	.808				
Acc 3	.521	.537			.777	.764				
Acc 4	.488	.363			.638	.758				
(4) Law										
L 1	.345	.315					.778	.790		
L 2	.373	.383					.735	.816		
L 3	.398	.386					.767	.818		
L 4	.308	.358					.777	.597		
(5) Human resources										
HR 1	.409	.432							.727	.666
HR 2	.458	.614							.734	.636
HR 3	.398	.462							.746	.679
HR 4	.336	.477							.785	.604

*Note.* Coefficients for t1 were obtained from model 4a, all factor loadings are significant (*p* < .05)*.*

For the remaining analyses, we had planned to use the best fitting structural model. Looking at the fit indices for Models 1–4, Model 4 showed a slightly higher CFI and clearly the lowest SRMR compared to the other models. In addition, low factor correlations suggested particularly well-distinguished first-order factors. Therefore, we picked the nested Marsh/Shavelson model (Model 4) as the basic structural model to analyze the structural development of self-concept.

### Structural Change of Students’ Self-Concept

To investigate structural change, we first cross-validated the nested Marsh/Shavelson model with respect to the two waves of measurement. We began by specifying the model for t1 and t2 data with correlations of generic field-of-study-specific—i.e., business administration (BA)—self-concept and for subject-specific self-concepts across measurement points (Model 4a). Fit indices for this and the following models are summarized in Table 3. The model fit for Model 4a was acceptable. As depicted in Table 2, most factor loadings at t1 were similar to factor loadings at t2. As expected, loadings for mathematical subjects mostly decreased slightly, whereas loadings for verbal subjects mostly increased slightly. First-order factor correlations were higher at t1 compared to t2, and significant (all *p* < .05; see Figure 5).

**Table 3:** Model Fit of Measurement Invariance for Model 4 (Nested Marsh/Shavelson Model) across Anticipated and Actual Self-Concept

Model	χ^2^(*df*)	Δχ^2^(*df*)	AIC	CFI	ΔCFI	RMSEA	95% CI RMSEA	ΔRMSEA	SRMR	ΔSRMR
4a	1070.278 (591)*		15731.762	0.942		0.049	[0.044, 0.053]		0.081	
4b	1135.257 (622)*	64.979 (31)*	15734.741	0.938	-0.004	0.049	[0.045, 0.054]	0.000	0.083	+0.002
4c	1179.828 (642)*	44.471 (20)*	15739.312	0.935	-0.003	0.050	[0.045, 0.054]	+0.001	0.085	+0.002
4d	1333.468 (661)*	153.640 (19)*	15854.952	0.918	-0.027	0.055	[0.050, 0.059]	+0.005	0.093	+0.008

*Note.* Model 4a = nested Marsh/Shavelson model for t1 and t2 with correlations between the same constructs at t1 and t2; Model 4b = Model 4a with factor loadings and structural weights constrained to be equal across measurement points; Model 4c = Model 4b with intercepts constrained to be equal across measurement points; Model 4d = Model 4c with residual variances constrained to be equal across measurement points; χ^2^ = chi-square goodness-of-fit, df = degree of freedom; AIC = Akaike information criterion; CFI = comparative fit index; RMSEA = root mean square error of approximation; SRMR = standardized root mean square residual.

*p < .05.

Next, we tested structural invariance by fixing loadings (weak invariance, Model 4b), loadings and intercepts (strong invariance, Model 4c), and loadings, intercepts, and residual variances (Model 4d) to be equal across measurement points. Model comparisons based on changes in fit indices supported the assumption of both metric and scalar, but not strict, invariance (see Table 3).

Finally, to investigate whether subject-specific self-concepts are derived from a generic self-concept or vice versa, we replicated analyses suggested by Marsh and Yeung (1998). Fit indices are summarized in Table 1 (Model 5–9). The baseline model was Model 5 with a horizontal effect of generic BA self-concept only. As indicated by the SRMR, fixing all but the direct effect of generic BA self-concept to zero apparently produces misfit in the model. Hence, the next model allowed for a direct horizontal effect of generic BA self-concept and of all subject-specific self-concepts (Model 6). This model showed an acceptable overall model fit. Interestingly, t1 generic BA self-concept significantly predicted t2 generic BA self-concept (β = .524, *p* < .05), t1 accounting self-concept significantly predicted t1 accounting self-concept (β = .569, *p* < .05), and t1 arithmetic self-concept significantly predicted t2 arithmetic self-concept (β = .593, *p* < .05), whereas law (β = .036) and human resources (β = .012) self-concepts at t1 did not show significant effects on the respective t2 self-concepts.

Next, we tested top-down effects of generic BA self-concept only (broken lines in Figure 5; Model 7). Results revealed a significant effect of t1 generic BA self-concept on t2 accounting self-concept (β = .176, *p* < .05). The remaining effects were non-significant (arithmetic: β = .058; law: β = -.162; HRM: β = .057). Similarly, the test for bottom up effects of subject-specific self-concepts at t1 on generic BA self-concept at t2 (dotted lines in Figure 5, Model 8) revealed a significant effect only for accounting (β = .229, *p* < .05), whereas all other effects were non-significant (arithmetic: β = .004; law: β = -.022; human resources: β = .039). In addition, model fit suggested a worse fit of Model 7 and 8 compared to Model 6. Finally, we tested a model that allowed for top-down, bottom-up, and horizontal effects simultaneously (Model 9). As depicted in Figure 5, we found horizontal effects as revealed in Model 6. By contrast, this model did not reproduce the top-down and bottom-up effects between generic BA self-concept and accounting self-concepts. In addition, the negative effect of t1 generic BA self-concept on t2 law self-concept reached significance.

## Discussion

The present study examined the structure and structural change of first-year students’ study-related self-concept using a longitudinal dataset (t1 at the beginning of study program; t2 toward the end of the first semester). A first-order factor model, a Shavelson model, a Marsh/Shavelson model, and a nested Marsh/Shavelson model all showed an acceptable fit to the data. Nevertheless, the nested Marsh/Shavelson model captured large proportions of shared variance in a generic field-of-study-specific self-concept factor specified as method factor (Eid et al., 2003), leading to well-distinguished subject-specific factors. Building on the nested Marsh/Shavelson model, further analyses revealed strong structural invariance. Further, results revealed significant horizontal effects for generic field-of-study-specific self-concept (i.e., the method factor) as well as for self-concepts related to the subjects accounting and arithmetic, but only one negative top-down effect of t1 generic field-of-study-specific self-concept on the self-concept related to the subject law. Our results did not show any bottom-up effects. Thus, our results suggest an independent development of generic field-of-study-specific and subject-specific self-concepts.

### Placing Generic Field-of-Study-Specific Self-Concept in Relation to Subject-Specific Self-Concepts

Our results support the assumption of both multidimensionality and hierarchy regarding self-concept structure when students face novel academic learning content. With respect to the Shavelson model, factor loadings on one second-order factor were relatively low, especially for verbal subjects, even though in our study—unlike languages in Yeung et al.’s (1999) study—all subjects were clearly field-of-study-related. The Marsh/Shavelson model could capture more domain-specific variance, but showed a high correlation between verbal and mathematical self-concept, which indicated shared variance unaccounted for by the model. Following the call for a higher-order factor that captures as much variance as possible shared by the first-order factors, the nested Marsh/Shavelson model incorporating generic field-of-study-specific self-concept as a method factor clearly performed best in this task, indicated by mostly non-significant first-order factor correlations. Hence, our results further support the validity of the nested self-concept structure proposed by Brunner et al. (2010).

Our study differed in the conceptualization of the second-order self-concept factor in that we proposed a generic, but field-of-study-specific, self-concept factor rather than a generic academic self-concept to be at the apex of the hierarchy. Supporting this proposition, results from the Shavelson model showed an almost perfect correlation between a second-order factor indicated by first-order subject-specific factors and a generic field-of-study-specific self-concept factor indicated by items referring to the field of study directly. This finding challenges Yeung et al.’s (1999) argument stating that a global academic self-concept conceptualized superordinate to subject-specific self-concepts can be found in educational contexts with a distinct focus. Rather, because Yeung et al. (1999) measured academic self-concept in relation to the students’ current educational context (i.e., a school of commerce), their academic self-concept measure may be considered identical to a generic commerce-specific self-concept (i.e., analogous to our generic business administration-specific self-concept in a sample of business adminstration students). Thus, we argue that in educational settings with a distinct focus, the hierarchy is established because all subjects relate to a specific domain or field of study, which may be similar to artistic self-concept (Vispoel, 1995), for example. From our results, this assumption appears to be valid although business administration covers a broad range of different subjects.

### Structural Change of Self-Concept During the Initial Study Phase

The present study was set at the beginning of students’ study program because we assumed substantial structural variability during the initial study phase. However, although some parameters changed somewhat, the overall structure of the nested Marsh/Shavelson model was invariant with respect to both factor loadings and intercepts. The most notable changes occurred regarding factor correlations, which were substantial at the beginning of the study program, but markedly lower and mostly non-significant toward the end of the first semester. In line with expectations, these results point to a better alignment of subject-specific self-concepts under the roof of a generic field-of-study-specific self-concept once students have made personal experiences with the subject matters in the context of their study program (labeled revision by Gorges & Kandler, 2012).

Regarding directional effects between generic field-of-study-specific self-concept and subject-specific self-concepts, we found but one (negative) top-down effect on subject-specific (i.e., law-specific) self-concept. Hence, as has been reported by Marsh and Yeung (1998), self-concepts are rather stable on their level of specificity, suggesting a relatively independent development of self-concept on the different levels of specificity. As this study was set early in students’ course of study, more top-down effects may be revealed at the beginning of the following semesters when novel subjects enter students’ schedule yet again, but by then, the students have a better idea of their field of study than at the very beginning of the study program. In the future, such longitudinal studies could offer further insight into self-concepts’ structural development. In addition, it may be worthwhile to extend the levels of specificity to, for example, topics within subjects (Dietrich, Viljaranta, Moeller, & Kracke, 2017). Subject-specific self-concepts appear to gain stability rather quickly, whereas topical self-concepts may still be malleable and may be informed more by subject-specific self-concepts than subject-specific self-concepts by academic or field-of-study-specific self-concept. In addition, linking self-concepts on different levels of specificity to relevant outcome variables, such as academic achievement, will be important tasks to be addressed in the future.

#### Implications for Self-Concept Research

The majority of self-concept research has focused on the level of school subjects, or on the two higher-order factors proposed by Marsh (1990). Beyond that, a handful of studies have addressed the position of academic self-concept at the apex of the self-concept hierarchy (Byrne & Gavin, 1996; Yeung et al., 2000). However, self-concept—defined as beliefs about one’s ability—is not limited to these analytical levels. Rather, self-concept may be addressed based on different structures provided by students’ educational context, for example, fields of study, specialized upper secondary schools, or even vocational and further education contexts. Zooming in, self-concepts may also relate to smaller entities of learning content than school or university subjects (e.g., Dietrich et al., 2017). Hence, we suggest that self-concept research could benefit from a broader view of self-concept structures.

Analogous to research on self-concept hierarchy, its structural development has hardly been addressed. Therefore, our second proposition for future self-concept research is that more attention should be paid to processes of self-concept formation and structural development when students face novel academic tasks in particular. Although we focused on students’ entering college, such processes should also be found during secondary school, when new subjects are added to the curriculum, as well as in vocational and further education contexts. These areas of academic self-concept have been neglected but should receive more attention in future research. In addition, perceptions of ability depend on a range of factors (e.g., past achievement, reference groups; Marsh, 2007) and their effect of performance and task choice probably interacts with perceptions of social norms and expectations (e.g., stereotypes about what a girl can or cannot do well; Schmader, Johns, & Forbes, 2008). Therefore, self-concept research could benefit from taking into account mechanisms outside of already well-established developmental models as well.

#### Practical Implications

The results of the present study suggest that first-year students anticipate differential study-related self-concepts both on the level of field of study and on the level of singular subjects that are part of the study program. The initial structure of their self-concept shows some variability during the initial study phase, but does not change fundamentally. Importantly, development of subject-specific and field-of-study-specific self-concepts appears to be relatively independent. Hence, teachers could help students to develop appropriate and—if possible—positive self-concepts by providing information on the competences attached to the various subjects that are part of the students’ study program (i.e., setting learning goals) and giving students frequent opportunities for practice and informative feedback (e.g., tutorials, exercises). Building and stabilizing students’ self-concept from day one may help them to overcome initial insecurity with respect to their aptitude for the study program, which, in turn, may prevent student attrition (Stinebrickner & Stinebrickner, 2009). A deliberate promotion of self-concept could thus be a bridge between ability perceptions that students bring with them when they face novel learning content (e.g., based on college aptitude tests or self-assessments), and the first round of exams, which may not be before the end of students’ first year in a new educational context.

#### Limitations and Outlook

The present study is the first to examine students’ study-related self-concept structure and structural change when they face novel academic learning content. Following seminal work by Marsh, Brunner, and their colleagues, we used well-established measures to assess self-concepts and sophisticated statistical approaches for our analyses. Nevertheless, the study has some important limitations that need to be considered in its interpretation.

First, compared to typical studies on self-concept structure (e.g., Arens & Jansen, 2015; Marsh, 1990), our sample may be considered relatively small. In addition, it has been recruited from a specific educational context and field of study, namely study programs labeled business administration at universities of applied sciences in Germany. Hence, the results need replication using different samples and educational contexts to further support claims for generalization. In particular, to establish the novelty of the learning content, the present study focused on a study program that did not relate to a school subject from the high school curriculum. It would be interesting to see if results were any different in a study focusing on a study program that has the same label as a school subject (e.g., mathematics, biology). In addition, universities of applied sciences typically have a substantial proportion of students’ with study-related practical experience (Willich, Buck, Heine, & Sommer, 2011), who may already have a more accurate notion of the subjects related to business administration (but see Gorges, 2017).

Second, the present study included self-concept measures only. While measuring self-concepts necessarily draws on self-report measures, the study lacks external criteria, such as subsequent achievement or task choice. Given that the relation of self-concept to other constructs (i.e., its “*empirical network*”; Brunner et al., 2010, p. 964, italics in original) is the second-most important component of self-concept research, future studies should incorporate achievement and/or task choice into their study design.

## References

[r1] Arens A. K. Jansen M. (2015). Self-concepts in reading, writing, listening, and speaking: A multidimensional and hierarchical structure and its generalizability across native and foreign languages. *Journal of Educational Psychology*, 108(5), 646–664. doi: 10.1037/edu0000081

[r2] Bong M. Skaalvik E. M. (2003). Academic self-concept and self-efficacy: How different are they really? *Educational Psychology Review*, 15(1), 1–40. doi: 10.1023/A:1021302408382

[r3] Brunner M. Keller U. Dierendonck C. Reichert M. Ugen S. Fischbach A. Martin R. (2010). The structure of academic self-concepts revisited: The nested Marsh/Shavelson model. *Journal of Educational Psychology*, 102(4), 964–981. doi: 10.1037/a0019644

[r4] Byrne B. M. Gavin D. A. (1996). The Shavelson Model revisited: Testing for the structure of academic self-concept across pre-, early, and late adolescents. *Journal of Educational Psychology*, 88(2), 215–228. doi: 10.1037/0022-0663.88.2.215

[r5] Chen F. F. (2007). Sensitivity of goodness of fit indexes to lack of measurement invariance. *Structural Equation Modeling*, 14(3), 464–504. doi: 10.1080/10705510701301834

[r6] Cheung G. W. Rensvold R. B. (2002). Evaluating goodness-of-fit indexes for testing measurement invariance. *Structural Equation Modeling*, 9(2), 233–255. doi: 10.1207/S15328007SEM0902_5

[r7] Denissen J. J. A. Zarrett N. R. Eccles J. S. (2007). I like to do it, I’m able, and I know I am: Longitudinal couplings between domain-specific achievement, self-concept, and interest. *Child Development*, 78(2), 430–447. doi: 10.1111/j.1467-8624.2007.01007.x 17381782

[r8] Dickhäuser O. S. Schöne C. C. Spinath B. Stiensmeier-Pelster J. (2002). Skalen zum akademischen Selbstkonzept: Konstruktion und Überprüfung eines neuen instruments. [The academic self concept scales: Construction and evaluation of a new instrument]. *Zeitschrift für Differentielle und Diagnostische Psychologie*, 23(4), 393–405. doi: 10.1024//0170-1789.23.4.393

[r9] Dietrich J. Viljaranta J. Moeller J. Kracke B. (2017). Situational expectancies and task values as predictors of students’ effort. *Learning and Instruction*, 47, 53–64. doi: 10.1016/j.learninstruc.2016.10.009

[r10] Eid M. Lischetzke T. Nussbeck F. W. Trierweiler L. I. (2003). Separating trait effects from trait-specific method effects in multitrait-multimethod models: A multiple-indicator CT-C (M-1) model. *Psychological methods*, 8(1), 38–60. doi: 10.1037/1082-989X.8.1.38 12741672

[r11] Elliot, A. J., & Dweck, C. S. (2005). Competence and motivation. Competence as the core of achievement motivation. In A. J. Elliot, & C. S. Dweck (Eds.), *Handbook of competence and motivation* (pp. 3–12). New York, NY, USA: Guilford Press.

[r12] Gorges J. (2017). First-year students’ initial motivational beliefs at university. Predicted by motivational beliefs derived from within and out-of-school experience and malleable regardless of the extent of students’ out-of-school experience. *Frontiers in Psychology*, 8, 1258. doi: 10.3389/fpsyg.2017.01258 28790951 PMC5524895

[r13] Gorges J. Goeke T. (2015). How do I know what I can do? Anticipating expectancy of success regarding novel academic tasks. *British Journal of Educational Psychology*, 85(1), 75–90. doi: 10.1111/bjep.12064 25557305

[r14] Gorges J. Kandler C. (2012). Adults’ learning motivation: Expectancy of success, value, and the role of affective memories. *Learning and Individual Differences*, 22(5), 610–617. doi: 10.1016/j.lindif.2011.09.016

[r15] Jansen M. Schroeders U. Lüdtke O. Pant H. A. (2014). Interdisziplinäre Beschulung und die Struktur des akademischen Selbstkonzepts in den naturwissenschaftlichen Fächern [Interdisciplinary science classes and the dimensional structure of academic self-concept in the sciences]. *Zeitschrift für Pädagogische Psychologie*, 28(1–2), 43–49. doi: 10.1024/1010-0652/a000120

[r16] Lau I. C. Y. Yeung A. S. Jin P. Low R. (1999). Toward a hierarchical, multidimensional English self-concept. *Journal of Educational Psychology*, 91(4), 747–755. doi: 10.1037/0022-0663.91.4.747

[r17] Leonard N. H. Beauvais L. L. Scholl R. W. (1999). Work motivation: The incorporation of self-concept-based processes. *Human Relations*, 52(8), 969–998. doi: 10.1177/001872679905200801

[r18] Marsh H. W. (1990). The structure of academic self-concept: The Marsh/Shavelson model. *Journal of Educational Psychology*, 82(4), 623–636. doi: 10.1037/0022-0663.82.4.623

[r19] Marsh H. W. (1992). Content specificity of relations between academic achievement and academic self-concept. *Journal of Educational Psychology*, 84(1), 35–42. doi: 10.1037/0022-0663.84.1.35

[r20] Marsh, H. W. (2007). *Self-concept theory, measurement and research into practice: The role of self-concept in educational psychology.* Leicester: British Psychological Society.

[r21] Marsh H. W. Byrne B. M. Craven R. (1992). Overcoming problems in confirmatory factor analyses of MTMM data: The correlated uniqueness model and factorial invariance. *Multivariate Behavioral Research*, 27(4), 489–507. doi: 10.1207/s15327906mbr2704_1 26811131

[r22] Marsh H. W. Craven R. G. (2006). Reciprocal effects of self-concept and performance from a multidimensional perspective: Beyond seductive pleasure and unidimensional perspectives. *Perspectives on Psychological Science*, 1(2), 133–163. doi: 10.1111/j.1745-6916.2006.00010.x 26151468

[r23] Marsh H. W. Hey J. Johnson S. Perry C. (1997). Elite athlete self-description questionnaire: Hierarchical confirmatory factor analysis of responses by two distinct groups of elite athletes. *International Journal of Sport Psychology*, 28(3), 237–258. Retrieved from http://psycnet.apa.org/psycinfo/1997-38935-003

[r24] Marsh, H. W., Martin, A. J., Yeung, A. S., & Craven, R. (2017). Competence self-perceptions. In A. J. Elliot, C. S. Dweck, & Yeager (Eds.), *Handbook of competence and motivation* (2nd ed.) (pp. 85–115). New York, NY, USA: Guilford Press.

[r25] Marsh H. W. Yeung A. S. (1998). Top-down, bottom-up, and horizontal models: The direction of causality in multidimensional, hierarchical self-concept models. *Journal of Personality and Social Psychology*, 75(2), 509–527. doi: 10.1037/0022-3514.75.2.509 9731322

[r26] Meredith W. (1993). Measurement invariance, factor analysis and factorial invariance. *Psychometrika*, 58(4), 525–543. doi: 10.1007/BF0229482

[r27] Musu-Gillette L. E. Wigfield A. Harring J. R. Eccles J. S. (2015). Trajectories of change in students’ self-concepts of ability and values in math and college major choice. *Educational Research and Evaluation*, 21(4), 343–370. doi: 10.1080/13803611.2015.1057161

[r28] Muthén, L. K., & Muthén, B. O. (1998–2016). *Mplus users’ guide version 7.0*. Los Angeles, CA, USA: Muthén & Muthén.

[r29] Schafer J. L. Graham J. W. (2002). Missing data: Our view of the state of the art. *Psychological Methods*, 7(2), 147–177. doi: 10.1037/1082-989X.7.2.147 12090408

[r30] Schermelleh-Engel K. Moosbrugger H. Müller H. (2003). Evaluating the fit of structural equation models: Tests of significance and descriptive goodness-of-fit measures. *Methods of Psychological Research Online*, 8(2), 23–74.

[r31] Schmader T. Johns M. Forbes C. (2008). An integrated process model of stereotype threat effects on performance. *Psychological Review*, 115(2), 336. doi: 10.1037/0033-295X.115.2.336 18426293 PMC2570773

[r32] Shavelson R. J. Hubner J. J. Stanton G. C. (1976). Self-concept: Validation of construct interpretations. *Review of Educational Research*, 46(3), 407–441. doi: 10.3102/00346543046003407

[r33] Stinebrickner, T. R., & Stinebrickner, R. (2009). *Learning about academic ability and the college drop-out decision* (No. w14810). Cambridge, MA, USA: National Bureau of Economic Research.

[r34] Tempelaar D. T. Gijselaers W. H. van der Loeff S. S. Nijhuis J. F. (2007). A structural equation model analyzing the relationship of student achievement motivations and personality factors in a range of academic subject-matter areas. *Contemporary Educational Psychology*, 32(1), 105–131. doi: 10.1016/j.cedpsych.2006.10.004

[r35] Vispoel W. P. (1995). Self-concept in artistic domains: An extension of the Shavelson, Hubner, and Stanton (1976) model. *Journal of Educational Psychology*, 87(1), 134–153. doi: 10.1037/0022-0663.87.1.134

[r36] Willich, J., Buck, D., Heine, C., & Sommer, D. (2011). *Studienanfänger im Wintersemester 2009/10. Wege zum Studium, Studien- und Hochschulwahl, Situation bei Studienbeginn*. [First-year students in winter term 2009/10. Avenues into studying, choices of study program and higher education institution, situation at the start of the study program]. Hannover, Germany: HIS.

[r37] Yeung A. S. Chui H. S. Lau I. C. Y. (1999). Hierarchical and multidimensional academic self-concept of commercial students. *Contemporary Educational Psychology*, 24(4), 376–389. doi: 10.1006/ceps.1998.0994 10508533

[r38] Yeung A. S. Chui H. S. Lau I. C. Y. McInerney D. M. Russell-Bowie D. Suliman R. (2000). Where is the hierarchy of academic self-concept? Journal of Educational Psychology, 92(3), 556–567. doi: 10.1037/0022-0663.92.3.556

